# Refractive Alterations in Marfan Syndrome: A Narrative Review

**DOI:** 10.3390/medicina61020250

**Published:** 2025-02-01

**Authors:** Dionysios G. Vakalopoulos, Stamatios Lampsas, Marina S. Chatzea, Konstantina A. Togka, Vasileios Tsagkogiannis, Dimitra Mitsopoulou, Lida Lalou, Aikaterini Lampsa, Marios Katsimpras, Petros Petrou, George D. Kymionis

**Affiliations:** 1 1st Department of Ophthalmology, “G. Gennimatas” Hospital, Medical School, National and Kapodistrian University of Athens, 11528 Athens, Greece; dionisis.vakalopoulos@gmail.com (D.G.V.); marinachatzea@gmail.com (M.S.C.); constantinatoga@gmail.com (K.A.T.); btsago@gmail.com (V.T.); dimits96@gmail.com (D.M.); katsimpras_marios@hotmail.com (M.K.); petrospetroueye@gmail.com (P.P.); 2 2nd Department of Ophthalmology, Attikon University Hospital, Medical School, National and Kapodistrian University of Athens, 11528 Athens, Greece; lampsas.stam@gmail.com (S.L.); katerinalam2000@hotmail.com (A.L.)

**Keywords:** Marfan syndrome, refractive alterations, ectopia lentis, refractive management, ocular manifestations

## Abstract

Marfan syndrome (MFS) is a genetic disorder that affects the connective tissue in several systems, with ocular, cardiovascular, and skeletal system manifestations. Its ocular manifestations include ectopia lentis (EL), myopia, astigmatism, and corneal abnormalities. This review examines refractive alterations related to MFS such as EL, microspherophakia, lens coloboma, altered corneal biomechanics (flattening, thinning, and astigmatism), and myopia and their impact on visual acuity. The pathogenesis of these manifestations stems from mutations in the FBN1 gene (encoding fibrillin-1). Moreover, the current medical and surgical management strategies for MFS-related refractive errors, including optical correction (eyeglasses, contact lenses, etc.), and surgical interventions like lensectomy, intraocular lens (IOL) implantation (anterior chamber, posterior chamber, scleral-fixated, iris-fixated), and the use of capsular tension rings/segments are further discussed. Considering the likelihood of underdiagnosing and underestimating ocular involvement in MFS, this updated review highlights the critical need to identify and address these refractive issues to enhance the visual outcomes for those affected.

## 1. Introduction

Marfan syndrome (MFS) is the most common genetic disorder of the connective tissue, which predominantly affects the ocular, cardiovascular, and skeletal systems, with an estimated prevalence of 1 in 10,000 [1]. Common ocular manifestations include ectopia lentis, abnormal corneal curvature, and increased axial length [2]. Its cardiovascular complications often involve aortic root aneurysms and valve dysfunction. Its skeletal features include arachnodactyly, tall stature, and pectus deformities [3,4]. The ocular manifestations of the disorder seem to be strongly related to cardiovascular comorbidities [5]. This condition arises from abnormalities in the production of fibrillin-1 (FBN1) [6].

The typical physical manifestations of MFS include excessively long bones, often accompanied by scoliosis, chest wall deformities, and greater joint flexibility [7]. Musculoskeletal issues are frequently the earliest signs that prompt consideration of an MFS diagnosis [8]. The Ghent nosology criteria, first introduced in 1996 and revised in 2010, are the clinical guidelines for diagnosing MFS. The revised criteria place greater emphasis on aortic root dilation and ectopia lentis, as these are seen as primary diagnostic features of MFS. They also incorporate genetic testing for mutations in the FBN1 gene [9,10].

This review aims to elaborate on how MFS can lead to ocular involvement and refractive alterations, including ectopia lentis, increased axial length, zonular instability, iris transillumination defects, retinal detachment, decreased corneal curvature, and scleral thinning. Moreover, novel medical and surgical treatment approaches to the above ocular refractive abnormalities are discussed. Novel medical and surgical treatment approaches to the above ocular refractive abnormalities are discussed. Given that MFS may not be immediately suspected by clinicians and its effects on the eyes can be overlooked or underestimated, an updated review of the literature on MFS-related refractive manifestations is necessary.

## 2. Epidemiology

**Figure 1:**
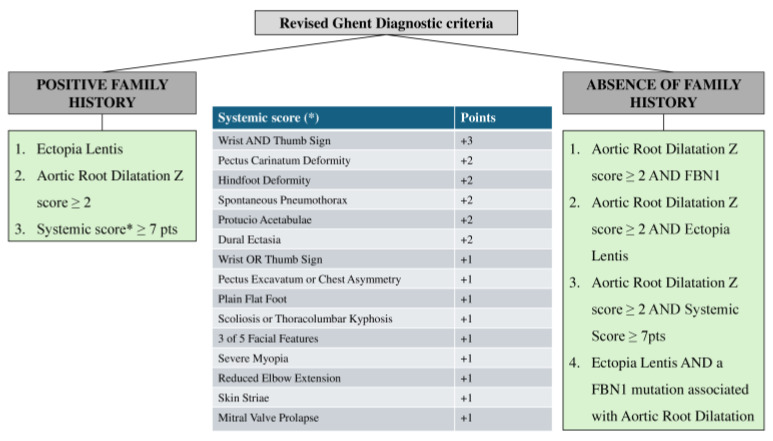
Revised Ghent diagnostic criteria for Marfan syndrome (MFS).

The prevalence of this disease was previously difficult to be estimate since firstly, there used to be no diagnostic criteria, and many cases without a characteristic phenotype remained undiagnosed or mistakenly diagnosed as MFS [3]. Initially, a biased prevalence of 1 per 4000 or 6000 was reported, which was based on patients in the Baltimore–Washington region. In 1986, the first diagnostic criteria were established in Berlin [11]. After their application in diagnosis, the following results arose: 1.5 per 100,000 people in Northern Ireland, 4.6 per 100,000 people in Denmark, and 6.8 per 100,000 people in Northeastern Scotland [12,13,14]. However, molecular techniques still did not exist, and these results may have been overestimated. In 1996, the Ghent I diagnostic criteria were established, and they included the FBN1 gene’s mutations for the first time [15]. In 2010, the Ghent criteria were revised, and from then on, the Ghent II diagnostic criteria have been used for MFS diagnosis [10] (Figure 1). In 2015, with the aid of these criteria, a prevalence of 6.5 per 100,000 people was reported in Denmark [3]. In 2018, a prevalence of 4.36 per 100,000 people was mentioned, and the most common age of diagnosis was between 10 and 19 years old. Ectopia lentis appeared in 21.7% of patients, and 43% of these cases were treated with surgery [16]. These numbers do not differ between the two sexes [11]. Recently, a Danish Nationwide Register Study reported a prevalence of 6.5 per 100,000 [17]. Some of the most commonly reported ocular manifestations among 3887 MFS patients were ectopia lentis, retinal tears or detachment, aphakia, pseudophakia, and high myopia, respectively [5]. Moreover, patients with ocular manifestations of MFS showed a significantly higher likelihood of being diagnosed with all modeled cardiovascular comorbidities, including aortic aneurysm and dissection [5]. Notably, according to a large registry, among MFS subjects, 56% had at least one recorded ophthalmic manifestation [17].

Some factors prevent or delay a diagnosis, leading to a reduced reported prevalence of the disorder. These factors are as follows: the phenotype becomes more characteristic with increasing age (delayed diagnosis), there is not a rapid molecular diagnostic test for FBN1 gene mutations, some manifestations of the disease are observed in phenotypes of other disorders too (missed diagnoses), and 25% of cases are sporadic, without a positive family history [18,19]. The prevalence of MFS is also affected by cases sparing FBN1 gene mutations, who are usually patients with transforming growth factor beta receptor 1 (TGFBR1) or 2 missense mutations [20].

## 3. Pathophysiology

Alterations in MFS are caused by the FBN1 gene, which codes for fibrillin-1 and is located on chromosome band 15q15-2 [21]. Most of these alterations are mutations which change just 1 of the 2871 amino acids that make up the protein [22]. These mutations typically occur in the Epidermal Growth Factor (EGF)-like domains of the protein. They often impact the cysteine residues or amino acids involved in calcium binding, leading to a weakened and abnormal connective tissue structure [23].

There is a subtype of MFS which is called Marfan-like syndrome or MFS type 2, and it is also inherited in an autosomal dominant manner but without alterations in the FBN1 gene [24]. In this type of MFS, mutations are found on chromosome 3p24.2-25 on the TGFBR2 gene, which codes for transforming growth factor beta (TGF-b) [25]. Research on mice has demonstrated that a lack of fibrillin-1 results in elevated TGF-b levels. It is believed that the excessive activation and signaling of TGF-b contributes to the various symptoms of MFS and diverse Marfan phenotypes [26]. TGF-b is a cytokine that arranges cellular proliferation, differentiation, and apoptosis and preserves homeostasis of the extracellular matrix [23]. When TGF-b is produced, it remains inactive as part of a protein complex, which binds to the microfibrils of the extracellular matrix. After stimulation, TGF-b is released, and it is bonded to its receptor and starts signal transduction [23]. Fibrillin-1 controls this signaling pathway, so decreased or altered forms of fibrillin-1 fail to sequester the large protein complex, leading to excessive TGF-b activation and signaling [27].

**Figure 2:**
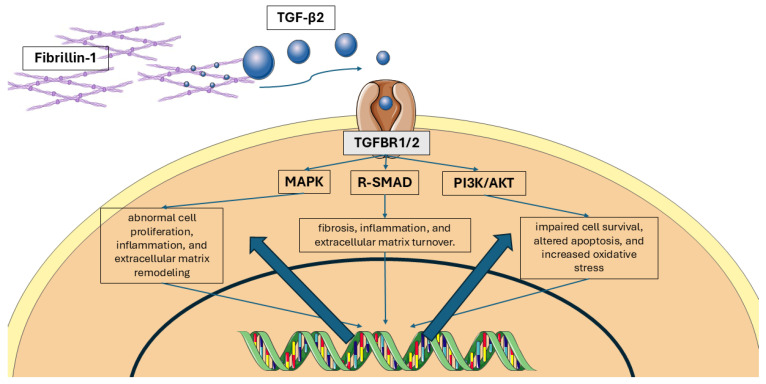
Marfan syndrome’s pathogenetic mechanism. Mutations in the fibrillin-1 (FBN1) gene disrupt the structure and function of the microfibrils, which are essential components of the extracellular matrix. This disruption leads to abnormal regulation of transforming growth factor beta (TGF-β) signaling and downstream pathways, including MAPK, PI3K, and R-SMAD signaling.

Fibrillin-1 is a glycoprotein that is widely found in many organs and systems throughout the body and provides elasticity and strength to the connective tissue and structural stability [3]. In the ocular system, fibrillin-1 is found in the following ocular structures: the ciliary zonules, the ciliary body, the lens’ capsule, the iris, the epithelial Bowman’s membrane, the endothelial Descemet’s membrane, the walls of Schlemm’s canal, the sclera, the corneal stroma, and the subepithelial region of the peripheral cornea [18,28]. Fibrillin-1 deficiency leads to ectopia lentis, miosis, hypoplasia of the iris, and refractive and corneal abnormalities [29] (Figure 2).

## 4. Lens Alterations

### 4.1. Ectopia Lentis

Ectopia lentis (EL) affects between 30.2% and 87.0% of patients with MFS, according to various studies [30,31,32]. Most recent studies report an average prevalence of 50% of ectopia lentis in individuals with MFS [33,34]. The dislocation typically occurs in the superotemporal direction. However, if the lens dislocates forward into the pupil or anterior chamber, it can lead to a pupillary block, which may result in acute glaucoma or chronic angle-closure glaucoma [35]. On the other hand, posterior dislocations can induce vitreoretinal traction, potentially causing chronic uveitis, chorioretinal inflammation, and an increased risk of retinal detachment [36]. Despite the wide variation in the incidence rates reported, EL remains a primary diagnostic criterion in the 2010 revised Ghent nosology for Marfan syndrome [14,37,38,39]. The role of the ciliary process in supporting the lens is essential in the development of lens luxation [39,40]. In normal human eyes, fibrillin is located on the surface of the ciliary epithelium, where the zonules attach, and within the ciliary muscle of the ciliary body [40]. Histologically, in normal human eyes, fibrillin is a critical component of the ciliary zonules and the superficial zone of the capsule, where the zonules are attached to the lens [40,41]. Studies have shown that individuals with MFS commonly had fewer zonules present in number and that they frequently appeared to be attenuated or broken [40]. Additionally, electron microscopy indicated that the zonular fibrils were thinner than normal and lacked the typical parallel alignment, exhibiting a haphazard arrangement instead [40]. Furthermore, proteolytic damage to the zonular microfibrils may further contribute to the pathogenesis of zonular dysfunction, leading to EL, while lens coloboma may also occasionally be observed [39,40,41].

### 4.2. Microspherophakia

Microspherophakia is characterized by a lens that is spherical in shape and has a reduced equatorial diameter [42]. Microspherophakia is highly associated with high refractive errors and lens dislocation [43]. The cortical fibers are about 20% of their normal thickness, indicating abnormal fibrogenesis rather than a complete halt in fiber development [44]. Genetic studies have linked autosomal dominant Weill–Marchesani syndrome (WMS)—WMS is a connective tissue disorder characterized by distinctive eye abnormalities, such as severe myopia, microspherophakia, and ectopia of the lenses—with MFS [45]. Both of these abnormalities show allelic disorders at the FBN1 locus, reflecting the clinical variability seen in type I fibrillinopathies [45].

### 4.3. Lens Coloboma

**Figure 3:**
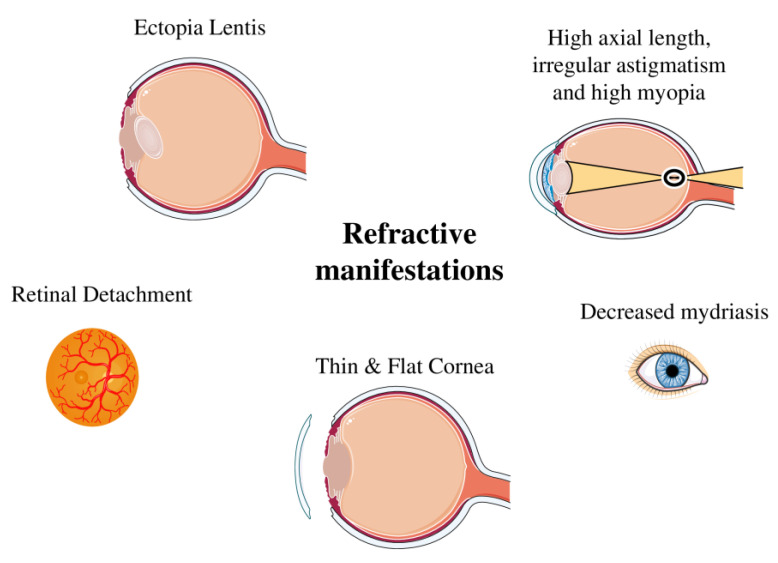
Refractive manifestations in Marfan syndrome (MFS).

Lens coloboma is a rare condition that can be associated with MFS and ectopia lentis. A lens coloboma is not a true coloboma (an absence of tissue) but rather an apparent defect caused by inadequate zonular support, leading to deformation of the lens’ shape [46]. In MFS, this can occur due to zonular fiber fragility, which is a hallmark of the syndrome. This can lead to various refractive errors such as myopia, irregular astigmatism, and hyperopia, due to an irregular lens shape or lens decentration [47]. However, this can cause symptoms of painless and progressively decreasing vision that demand challenging surgical interventions [47,48] (Figure 3).

## 5. Corneal Biomechanics

Flattened corneas have been reported as a complication in patients with MFS and are classified as a minor sign in the Ghent criteria [15,49]. A comparative study on 285 MFS patients and 267 controls found that most of the MFS patients had a flatter cornea than the eyes of the control group, with a mean keratometry (Km) below 41.5 D in at least one eye (Km 41.78 ± 1.80 diopters (D) versus 43.05 ± 1.51 D, *p* < 0.001) [50]. Corneal astigmatism was greater and the central cornea was thinner in eyes with MFS (530.14 ± 41.31 μm versus 547.02 ± 39.18 μm, *p* < 0.001), while eyes with MFS and EL exhibited both a flatter cornea (Km 41.13 ± 1.79 D versus 42.11 ± 1.66 D, *p* = 0.001) and higher corneal astigmatism (1.63 ± 1.15 D versus 1.00 ± 0.90 D, *p* < 0.001) than eyes with MFS without EL [50]. Another retrospective case–control study on 55 eyes in MFS patients with lens subluxation and 53 normal eyes in control subjects revealed the anterior and posterior corneal surfaces were significantly flatter in the MFS group than in the control group (both *p*  <  0.001) [51]. Regarding corneal astigmatism, anterior corneal astigmatism (ACA) and total corneal astigmatism (TCA) were significantly higher in the MFS group than in the control group (both *p*  <  0.001), whereas posterior corneal astigmatism (PCA) was not significantly different between the two groups [51]. The same study proposed that a posterior corneal curvature value less than 6.0 D may become an indicator of MFS [51]. Although the exact cause of the flattened cornea remains unclear, it could be related to the increased size of the globe in MFS patients [34]. The resulting decreased curvature of the cornea could be a pathological expression of the abnormal fibrillin located between collagen lamellae of the thinned sclera [52,53]. Alternatively, this pathology could originate from corneal misdevelopment due to fibrillin mutations. In normal corneas, fibrillin is primarily localized in the basement membrane of the corneal epithelium, with stronger expression in the peripheral cornea compared to the central cornea, as well as between the collagen lamellae of the corneal stroma [34]. FBN-1 may play a role in providing structural support within the cornea. Morphological abnormalities in the elastic components contribute to the centrifugal stretching of the cornea and sclera, potentially leading to ocular enlargement, flattening of the cornea, and conditions such as megalocornea or cornea plana [34,40,54].

Keratoconus has occasionally been suggested in case reports to occur alongside MFS [55]. However, no studies to date have demonstrated a clear association between these two conditions. Prior research indicates that eyes in MFS with significant corneal astigmatism do not exhibit eccentric steepening, ectasia, or keratoconus-like changes upon examination using a slit lamp, as well as examination of the corneal topography [56]. Recent studies, however, have documented a reduced corneal thickness in MFS [34,57], with many cases showing corneal thicknesses below 500 μm [56]. These corneas were uniformly thin, differing from the localized thinning typical of keratoconus. Although corneal flattening is a recognized feature of MFS, a reduced corneal thickness is not yet a diagnostic criterion [10]. A prospective observational clinical study found that corneal hysteresis and the corneal resistance factor (CRF) were decreased in MFS (Marfan syndrome CH: 9.45 ± 1.62; control CH: 11.24 ± 1.21, *p* = 0.01; Marfan syndrome CRF: 9.77 ± 1.65; control CRF: 11.03 ± 1.72; *p* = 0.01) [58], in contrast with a previous study which found these values decreased only in MFS patients with ectopia lentis present [59], indicating that the corneas in MFS feature increased deformation and decreased bending resistance.

## 6. Refractive Errors

### 6.1. Myopia

Myopia, which is the most prevalent ocular disorder and progresses rapidly in early childhood, is the second most common ocular manifestation in MFS following EL, with myopia over 3 diopters (D) affecting 34–44% of MFS patients compared to 4.8% in the general population, according to several studies [40,50,60,61]. Consequently, myopia greater than 3 diopters is included in the revised Ghent nosology criteria for an MFS diagnosis. However, since myopia is prevalent in the general population, it contributes only one point to the systemic score [10]. Patients with MFS typically exhibit a longer AL but flatter corneas, which can partially compensate for the increased AL, resulting in a lower refractive error of less than 3.0 diopters [15,38,62]. In MFS patients, the mean axial length is 24.9 mm, with those with EL showing a higher average (25.96 mm) than those without EL (23.39 mm) [40]. Studies investigating the age-related differences in the axial length (AL) among MFS patients have identified significant variations across age groups. In children aged 10 years or younger, the average AL is significantly shorter and comparable to that in control groups without MFS [61]. In contrast, adults with MFS demonstrate a significantly increased AL, indicating that these differences in AL may become more pronounced as the eye grows and develops through adolescence [61]. Additionally, it is shown that higher degrees of myopia in MFS patients indicate an elevated risk for EL and increased axial length [50].

### 6.2. Astigmatism

Several studies have demonstrated that high corneal astigmatism is a significant factor in the diagnosis of MFS [38,50,56,63]. Structural changes due to advanced EL and frequent zonule defects in MFS patients leading to lenticular astigmatism may contribute to increased corneal astigmatism. Additionally, mutations in the FBN1 gene are thought to impact the corneal connective tissues, further elevating the levels of corneal astigmatism [38,54,63]. Previous research has shown that corneal astigmatism is higher in MFS patients with EL compared to those without EL, and overall, MFS patients exhibit greater corneal astigmatism than healthy individuals [38,56,63]. A study examining the corneal astigmatism orientation across different age groups in MFS patients found that 74.9% of the eyes exhibited with-the-rule (WTR) astigmatism, 11.2% against-the-rule (ATR), and 13.9% oblique astigmatism [38]. As age increased, there was a significant shift from WTR to ATR or oblique astigmatism [38], aligning with the patterns observed in healthy eyes [64]. This age-related shift in astigmatism orientation is thought to be influenced by factors such as reduced eyelid tension, increased intraocular pressure, age-related changes in extraocular muscle tension, and alterations in corneal structure [64].

### 6.3. Visual Acuity

Multiple studies have demonstrated that patients with MFS have lower visual acuity compared to that in healthy control groups [52,56,60]. The ocular abnormalities commonly associated with MFS, such as myopia, astigmatism, anisometropia, ectopia lentis, and retinal issues, can lead to complications like anisometropia, aniseikonia, and amblyopia [38,50,60,61]. A clinical study on genetic ectopia lentis found that significant amblyopia was more likely when the dislocated lens was near the center of the pupil, with the most severe cases occurring when the lens edge was positioned 1.3 mm from the center, partially blocking the visual axis [65]. These results indicate that early surgical intervention should be considered for children with lens subluxation who are unresponsive to conservative treatment.

## 7. Laser Refractive Surgery

The FDA discourages performing laser refractive surgery in individuals with MFS. This is due to potential risks such as post-LASIK ectasia, impaired wound healing, unpredictable refractive outcomes, and the possibility of globe rupture [48,66]. Marfan syndrome patients typically have higher rates of myopia and lenticular astigmatism due to lens subluxation [40,50]. Significant lens subluxation precludes this option, as it could interfere with future IOL calculations if surgery becomes necessary. Moreover, corneal astigmatism is sometimes more pronounced, which might complicate refractive correction [50]. However, most individuals with MFS typically exhibit a normal corneal tomography and a central corneal thickness (CCT) exceeding 500 µm. A 10-year follow-up study of MFS patients revealed stable myopia and no increase in the prevalence of refraction greater than -3D, corneal thinning, or keratoconus [67]. Due to their ocular stability, many of these patients are considered eligible candidates for various types of refractive surgery [66]. However, if a femtosecond platform is utilized, the lowest possible suction settings should be applied given the fragility of the cornea (reduced CH and CRFs) and the sclera [59,66], possibly increasing the risk of subretinal hemorrhage or retinal detachment [66]. A subset may present with high myopia, making iris-fixated phakic intraocular lenses (pIOLs) a suitable option, particularly in those without significant crystalline lens changes or signs of iridodonesis. Singh et al. implanted bilateral toric implantable collamer lenses (ICLs) in a patient with MFS and lens coloboma, with excellent postoperative outcomes [48].

Femtosecond laser-assisted cataract surgery (FLACS) has been performed in patients with lens subluxation and MFS [68,69,70,71]. Unlike traditional phacoemulsification, the femtosecond laser does not rely on zonular support for counter-resistance and can create a precise circular anterior capsulorhexis [72,73]. In situations involving zonulopathy, where the counter-traction is insufficient to stabilize the lens’ nucleus, the techniques like hydrodissection or viscodissection used in standard phacoemulsification may pose risks to the remaining zonular fibers and the capsular bag itself [73,74].

## 8. Management

The management of refractive alterations in MFS primarily targets the symptoms of EL, including blurred and fluctuating vision, monocular diplopia, and irregular astigmatism [60].

Surgical intervention is considered when the optimal visual acuity cannot be achieved using glasses or contact lenses due to cataracts or lens-dislocation-associated pathologies [75]. The surgical management of EL in MFS is complex due to the structural fragility of the zonules, lens capsule, iris, and sclera, which makes the eye more vulnerable to surgical complications [76]. Moreover, calculating the correct intraocular lens (IOL) power in patients with MFS might be challenging due to difficulties in predicting the effective lens position (ELP) [77] and changes in the AL in young patients [78]. The precision of IOL power calculation relies not only on the accuracy of the preoperative biometric parameters, such as axial length (AL), keratometry (K), and anterior chamber depth (ACD), with measurement inaccuracies in these parameters contributing to 36%, 22%, and 42% of the errors, respectively [79]. Advances in the phacoemulsification techniques, IOL options, and IOL power calculation formulas have increased surgical success [60,80,81].

Lens extraction procedures, such as pars plana vitrectomy (PPV) combined with lensectomy, are commonly employed in EL cases involving MFS. This approach minimizes the zonular stress and trauma by removing the lens material via a posterior approach [82]. Studies on pars plana lensectomy and vitrectomy in Marfan syndrome patients have shown improved visual outcomes with a low rate of complications; however, retinal detachment has been observed in a small percentage of cases [82,83].

The post-lensectomy vision correction options include aphakic glasses, contact lenses, or IOL implantation [84]. Aphakic glasses are generally preferred for pediatric patients because they are safe and have consistent outcomes. However, IOL implantation, including anterior chamber IOLs (ACIOLs), posterior chamber IOLs (PCIOLs), and scleral-fixated IOLs, are alternatives primarily for adults. Scleral-fixated PCIOLs, though they are more technically challenging, have been found to provide stable visual acuity without the excessive IOL movement seen with ACIOLs, which can lead to complications such as glaucoma, corneal decompensation, and inflammation [85]. However, PCIOL implantation carries a high rate of long-term complications, particularly in pediatric patients without adequate capsular support, with common issues including retinal detachment and IOL decentration [86]. A retrospective case–control study of 158 eyes concluded that in pediatric patients with MFS undergoing lens removal surgery, complete capsular removal appears to be the safer choice to minimize the risk of retinal detachment [87].

Iris-fixated IOLs present another option, particularly for cases without sufficient capsular support. These can be positioned in the anterior or posterior chamber but come with risks such as iris atrophy, IOL decentration, and retinal detachment [85]. Studies comparing various IOL fixation methods have shown mixed outcomes. For example, a randomized case series including 49 patients with MFS found similar rates of endothelial cell loss between iris-fixated and scleral-fixated IOLs but higher complication rates, including IOL decentration, retinal detachment, and elevated IOP, in the scleral fixation group [85].

**Figure 4:**
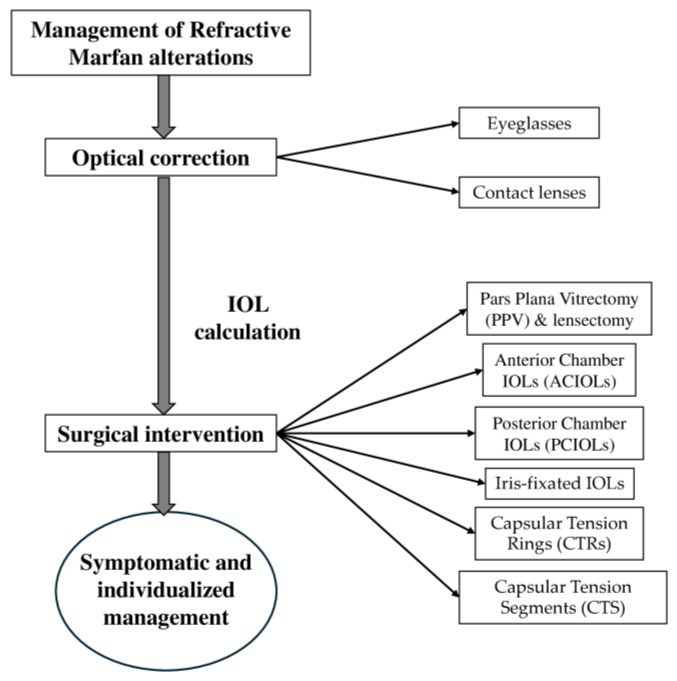
Medical and surgical management of refractive alterations in Marfan syndrome.

Capsular tension rings (CTRs) and capsular tension segments (CTSs) have been developed to stabilize the capsular bag during lens extraction and provide additional zonular support for IOL implantation. CTRs redistribute the zonular tension to the strongest remaining fibers, but they may not prevent further decentration as zonular degeneration progresses [60,88]. Cionni-modified CTRs, designed to be sutured to the sclera, and the capsular tension segment, which enables scleral fixation without rotation of the bag, provide enhanced support in cases with severe zonular weakness [89,90]. However, technical challenges can arise with the Cionni ring, particularly during capsulorhexis creation and implantation in highly unstable lenses, which may increase the risk of capsular bag tears. CTSs are beneficial due to their versatility, serving both as a support tool during surgery and as a fixation implant after the procedure. They are recommended in cases of significant zonular instability, usually when there is significant zonular dehiscence combined with phacodonesis [91] (Figure 4).

## 9. Conclusions

This review emphasizes that ocular manifestations, and particularly ectopia lentis, cataracts, and a high axial length, are often the earliest indicators of Marfan syndrome. These ocular abnormalities often precede systemic complications, making ophthalmologic assessments essential for its timely detection. Early diagnosis allows for prompt intervention, which can prevent more severe complications and improve the long-term outcomes.

The management strategies for ocular pathologies focus on preserving vision and improving quality of life. Ametropia, commonly associated with MFS, is typically addressed through corrective lenses. Cataract development, which can occur earlier in MFS patients, requires careful monitoring and timely surgical intervention to prevent further vision loss. Surgical approaches to ectopia lentis, when indicated, include lens removal and intraocular lens implantation, as tailored to the patient’s specific needs.

A multidisciplinary approach incorporating ophthalmologic, cardiologic, and genetic evaluations is crucial for the effective management of MFS, enabling clinicians to comprehensively address both its systemic and ocular challenges. Advancements in personalized strategies for managing refractive alterations in MFS have significantly enhanced therapeutic outcomes, offering a better quality of life for these patients.

## Data Availability

Not applicable.
